# Supplementary material for: Direct Current Stimulation Modulates Synaptic Facilitation via Distinct Presynaptic Calcium Channels
Source: Int J Mol Sci. 2023 Nov 28;24(23):16866. doi: 10.3390/ijms242316866 (PMC10706473; doi:10.3390/ijms242316866)
Supplement: Supplementary file 1 [file ijms-24-16866-s001.zip › ijms-2625077-supplementary.pdf]

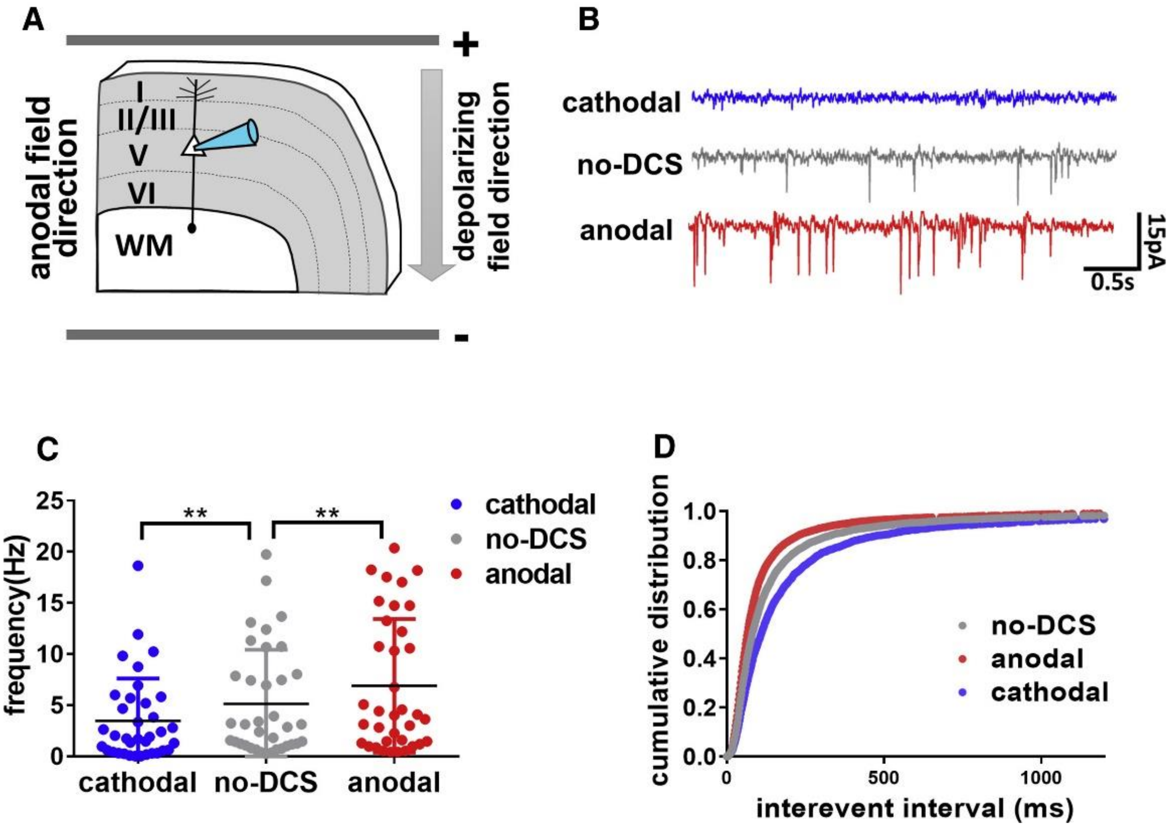

Supplementary Figure S1. DCS modulates the frequency of spontaneous excitatory postsynaptic currents (sEPSCs) recorded from layer-V pyramidal neurons at the M1 motor cortex. Anodal stimulation increases the sEPSCs frequency, while cathodal stimulation reduces the sEPSCs frequency.

Adapted from: Vasu O.V. and Kaphzan H., Cell Reports, 2021; 37(2):109832

(A) sEPSCs were recorded from soma of layer-V pyramidal neurons at the M1 motor cortex, when a DCS stimulation of 5V/m in the axonal-dendritic axis was applied across the slice in three polarizing DCS conditions: cathodal, no-DCS, and anodal. The illustration above (A) exclusively displays the anodal stimulation. The cortical layers situated both below and above the recorded layer-V are identified as Layer-I, representing the most superficial (upper), and layer-VI, signifying the deepest (lower).

(B) Sample traces of sEPSC recordings illustrate higher frequency and amplitude for anodal DCS condition (red) and lower frequency and amplitude for cathodal DCS conditions (blue) in comparison to no-DCS condition (gray).

(C) The average anodal frequency of sEPSC events are significantly larger than the no-DCS event frequency, whereas, the average cathodal frequency of sEPSC events are significantly smaller than the no-DCS event frequency ($F_{[1.40,48.96]} = 16.61$, $p < 0.0001$ in RM-ANOVA; $t_{(35)} = 3.4$, $p < 0.01$ in post hoc Bonferroni corrected comparison between anodal and no-DCS and $t_{(35)} = 3.6$, $p < 0.01$ in post hoc Bonferroni corrected comparison between cathodal and no-DCS) (n = 36 cells; N = 6 mice). Data are represented as mean ± SD. ** $p < 0.01$.

(D) Cumulative distributions of sEPSC inter-event intervals for cathodal and anodal conditions are significantly altered from no-DCS condition cumulative distribution. Anodal DCS induced a left shift of the cumulative distribution of inter-event intervals compared to the no-DCS distribution, whereas, cathodal DCS induced a right shift of the cumulative distribution of inter-event intervals compared to the no-DCS distribution ($D = 0.34$, $p < 0.0001$ in K-S between anodal and no-DCS and $D = 0.33$, $p < 0.0001$ in K-S between cathodal and no-DCS).
